# Breast Cancer Detection with an Ensemble of Deep Learning Networks Using a Consensus-Adaptive Weighting Method

**DOI:** 10.3390/jimaging9110247

**Published:** 2023-11-13

**Authors:** Mohammad Dehghan Rouzi, Behzad Moshiri, Mohammad Khoshnevisan, Mohammad Ali Akhaee, Farhang Jaryani, Samaneh Salehi Nasab, Myeounggon Lee

**Affiliations:** 1 School of Electrical and computer Engineering, College of Engineering, University of Tehran, Tehran 14174-66191, Iran; dehghanr.mohammad@gmail.com (M.D.R.); moshiri@ut.ac.ir (B.M.); akhaee@ut.ac.ir (M.A.A.); 2 Department of Electrical and Computer Engineering, University of Waterloo, Ontario, ON N2L 3G1, Canada; 3 College of Science, Northeastern University, Boston, MA 02115, USA; m.khoshnevisan@northeastern.edu; 4 Human Genome Sequencing Center, Baylor College of Medicine, Houston, TX 77030, USA; fjaryani@gmail.com; 5 Department of Computer Engineering, Lorestan University, Khorramabad 68151-44316, Iran; samaneh_salehi_nasab@yahoo.com; 6 College of Health Sciences, Dong-A University, Saha-gu, Busan 49315, Republic of Korea

**Keywords:** deep learning, mammograms, computer-aided detection, radiology, breast cancer, medical image analysis, ensemble learning, consensus-adaptive weighting

## Abstract

Breast cancer’s high mortality rate is often linked to late diagnosis, with mammograms as key but sometimes limited tools in early detection. To enhance diagnostic accuracy and speed, this study introduces a novel computer-aided detection (CAD) ensemble system. This system incorporates advanced deep learning networks—EfficientNet, Xception, MobileNetV2, InceptionV3, and Resnet50—integrated via our innovative consensus-adaptive weighting (CAW) method. This method permits the dynamic adjustment of multiple deep networks, bolstering the system’s detection capabilities. Our approach also addresses a major challenge in pixel-level data annotation of faster R-CNNs, highlighted in a prominent previous study. Evaluations on various datasets, including the cropped DDSM (Digital Database for Screening Mammography), DDSM, and INbreast, demonstrated the system’s superior performance. In particular, our CAD system showed marked improvement on the cropped DDSM dataset, enhancing detection rates by approximately 1.59% and achieving an accuracy of 95.48%. This innovative system represents a significant advancement in early breast cancer detection, offering the potential for more precise and timely diagnosis, ultimately fostering improved patient outcomes.

## 1. Introduction

Breast cancer remains the most frequently diagnosed cancer among women worldwide, leading to significant mortality. According to the World Health Organization (WHO), it accounted for 15% of all cancer-related deaths in women in 2018 [1]. The early detection of breast cancer through screening mammography can substantially reduce the mortality rate by 38–48% [2,3], and it has proven to be an effective tool for this purpose [4]. Consequently, numerous countries in the European Union are implementing screening programs to detect this disease at its early stages [5]. While imaging modalities like PET-CT (positron emission tomography–computed tomography) are also used, they are not recommended as primary diagnostic methods due to their high costs, relative invasiveness, and limitations in detecting small tumors [6,7]. This reaffirms the importance of mammograms in breast lesion classification.

Analyzing mammogram images is challenging due to the subtleties of early cancerous lesions and the variability in breast tissue density [8,9]. While essential, traditional screening by medical professionals has an error rate of around 30% [10,11,12], leading to potentially unnecessary biopsies and associated patient discomfort [13]. To mitigate these challenges, computer-aided detection (CAD) systems have been introduced to support radiologists [14]. When combined with human expertise, these systems can match the reliability of double human reading [15,16,17,18,19]. With the advent of artificial intelligence (AI), especially deep learning (DL) methods like convolutional neural networks (CNNs), the capability of CAD systems has been significantly enhanced [20,21,22,23,24,25,26,27,28]. Building on the transformative potential of AI in medical diagnostics, Bagheri et al. [29] exploited AI and ML techniques to tackle diagnostic challenges in chronic limb-threatening ischemia (CLTI), highlighting the utility of such methods for precise diagnoses, outcome predictions, and identifying treatment disparities. Furthermore, Park et al. [30] showcased another innovative application of machine learning, specifically the random forest approach, to remotely detect physical-aggressive episodes in children using wearable sensors, underscoring the expanding horizons of AI-driven medical insights, especially in behavioral contexts. With their capacity to derive hierarchical feature representations directly from data, DL models have shown promise in detecting intricate patterns in cancers, particularly breast cancer [31,32,33,34,35,36].

However, significant challenges remain in applying DL to mammogram interpretation. One major challenge is the size and complexity of mammogram images. Full-field digital mammography (FFDM) images are typically large, often around 4000 × 3000 pixels, with the area representing potential cancerous regions of interest (ROIs) being as small as 70 × 70 pixels [33]. This creates a proverbial “needle in a haystack” problem, where detecting small but clinically significant features is difficult. Region-based convolutional neural networks (R-CNN) offer one approach to addressing this issue by focusing on ROIs within the image [37,38,39]. However, they require pixel-level annotated data, which are labor-intensive and costly to generate. This limitation was highlighted in the study by Ribli et al. [33], which utilized the faster R-CNN [39] model for breast cancer detection but encountered challenges due to the small size of the pixel-level annotated dataset. Therefore, using region-based networks in medical images such as mammography is controversial.

Despite these challenges, majority voting has been widely used in machine learning tasks for decision fusion. Its simplicity and binary nature make it a common choice for ensemble models [40]. However, its binary nature limits its effectiveness, particularly in complex medical imaging tasks where the nuances of fuzzy logic can provide superior results.

With this research, we aim to contribute significantly to the evolution of CAD systems by introducing the CAW method as an ensemble of five deep learning methods. Our primary hypothesis posits that the performance of the CAW method will surpass that of any of the five individual networks. Furthermore, we hypothesize that our unique approach, which leverages fuzzy output instead of the traditional binary output, will enhance the system’s overall performance. This strategy not only addresses the challenges associated with pixel-level annotations but also bypasses the constraints of region-based networks. The ultimate goal of these advancements is to significantly enhance patient care and outcomes.

## 2. Materials and Methods

### 2.1. The Study Design

**Figure 1:**
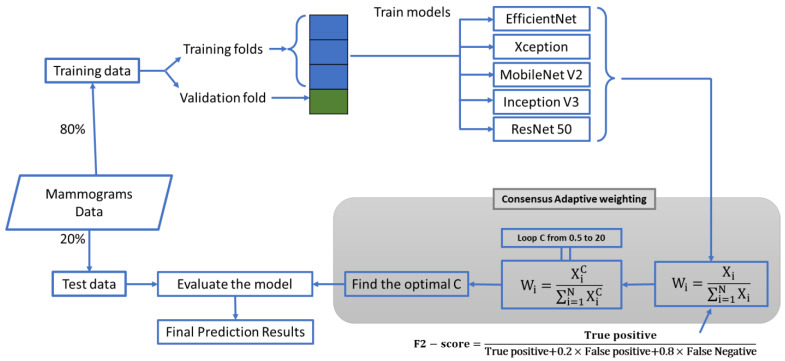
Flow chart of the CAD system. The grey area represents the proposed CAW model.

We proposed a fusion approach, leveraging the decision-making abilities of five different trained DL networks, namely EfficientNet [41], Xception [42], MobileNet V2 [43], Inception V3 [44], and Resnet50 [45]. Each of these networks has shown excellent performance in image classification tasks, complementing each other well in an ensemble setting [46,47,48,49]. This approach involved a consensus-adaptive weighting system based on the networks’ performance in detecting malignant mammograms. (Figure 1) Initially, we trained the five base networks (serving as the backbone of the ensemble model) on 80% of the dataset with four cross-validation folds. Following this, we calculated weights based on the performance of the trained networks using CAW Version 1 (V1) and Version 2 (V2) as we explained them in the following. Finally, we estimated the ensemble model’s performance using the test dataset.

### 2.2. The First Proposed CAW System

In medical diagnostic tasks such as breast cancer detection, maximizing recall is often more important than precision, as failing to detect a positive case could have dire consequences. Therefore, instead of the F1 score, which equally weights precision and recall, we proposed using the F2 score for our weighting system [50]. The F2 score places more emphasis on recall, aligning more closely with the priorities of image processing tasks. This method, named “consensus-adaptive weighting (CAW)”, is adaptive in nature, allowing for adjustment of weights when additional DL networks are incorporated. Although we included only five recent DL networks in our ensemble model, any number of networks could be incorporated at any time.

After training the five networks on 80% of the data, we employed CAW V1 (Equation (1)) as our ensemble model’s weighting system, and we used the F2 score (Equation (2)) to assign weights.

(1) $$W_{i}=\frac{X_{i}}{\sum_{i=1}^{N}X_{i}}$$

(2) $$F2-\mathrm{score}=\frac{\mathrm{True}\;\mathrm{positive}}{\mathrm{True}\;\mathrm{positive}+0.2\times \mathrm{False}\;\mathrm{positive}+0.8\times \mathrm{False}\;\mathrm{Negative}}$$

In Equation (1), $w_{i}$ stands for the weight of each model and $X_{i}$ stands for the F2 score of the trained network i. As we integrated more DL networks into our system, the weights $X_{i}$ were adaptively updated to reflect the contribution of each network in the ensemble.

### 2.3. The Second Proposed CAW System

Most of the time, the five DL networks’ performances are closely matched, making it challenging for CAW V1 to distinguish their weights effectively. As a solution, we proposed CAW V2 (Equation (3)), which resulted in a more significant differentiation in weights between networks. This method, shown to provide superior results in the results section, refines Equation (1).

(3) $$w_{i}=\frac{X_{i}{}^{C}}{\sum_{i=1}^{N}X_{i}{}^{C}}{\;\;\;\;\;\;\;\;\;\;\;\;\;\;\;X}_{i}=F2-\mathrm{score}\;\mathrm{of}\;\mathrm{the}\;\mathrm{trained}\;\mathrm{network}\;i$$

In Equation (3), ‘C’ represents the power ranging from 0.5 to 20 in increments of 0.1, determined empirically. To identify the optimal ‘C’, we conducted an exhaustive search across all cross-validation subsets of the dataset.

### 2.4. Dataset and Experiment Setup

To demonstrate the efficacy of our proposed model, we conducted tests on three publicly available mammogram datasets: Cropped DDSM (Digital Database for Screening Mammography) [51], DDSM [52], and INbreast [53]. Each dataset has been extensively used in the literature, emphasizing their relevance and reliability. However, due to the labor-intensive and time-consuming nature of compiling and labeling medical datasets, we restricted our study to these three datasets.

**Figure 2:**
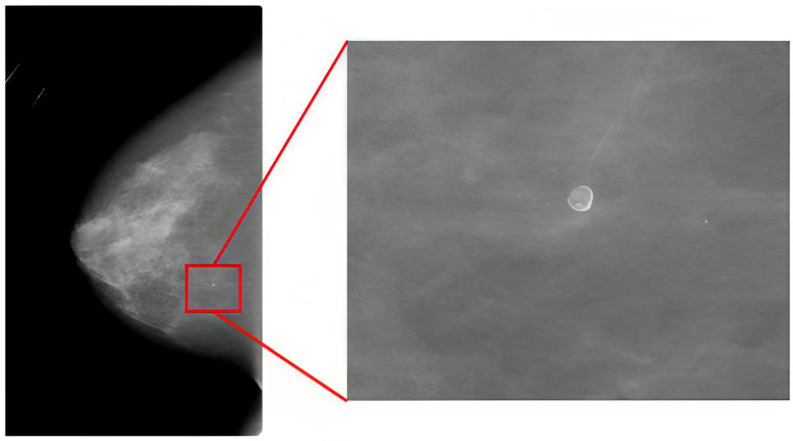
A sample mammogram image from the DDSM dataset is on the left, with a lesion in the middle, and on the right, there is a cropped image of the same lesion with a size of 299 × 299 that is part of the cropped DDSM dataset.

The cropped DDSM dataset comprises 55,890 training images, 14% of which are labeled as positive for breast cancer. Each image within this dataset measures 299 × 299 pixels (Figure 2). Given the relatively low proportion of positive cases (14%), we noted class imbalance in this dataset. To avoid imbalanced class problems [54], we randomly selected 7290 images from the 48,600 images labeled as negative for breast cancer. As a result, both classes were represented equally within the dataset (14,580 images in total).

The DDSM [52] dataset contains 7808 images, 35% marked as malignant (Figure 2). Given the lower overall number of cases in this dataset, we achieved class balance by randomly equalizing classes within each batch during the training process. Additionally, we resized the original 3000 × 4000 pixel images to 400 × 250 pixels as part of the training regimen.

**Figure 3:**
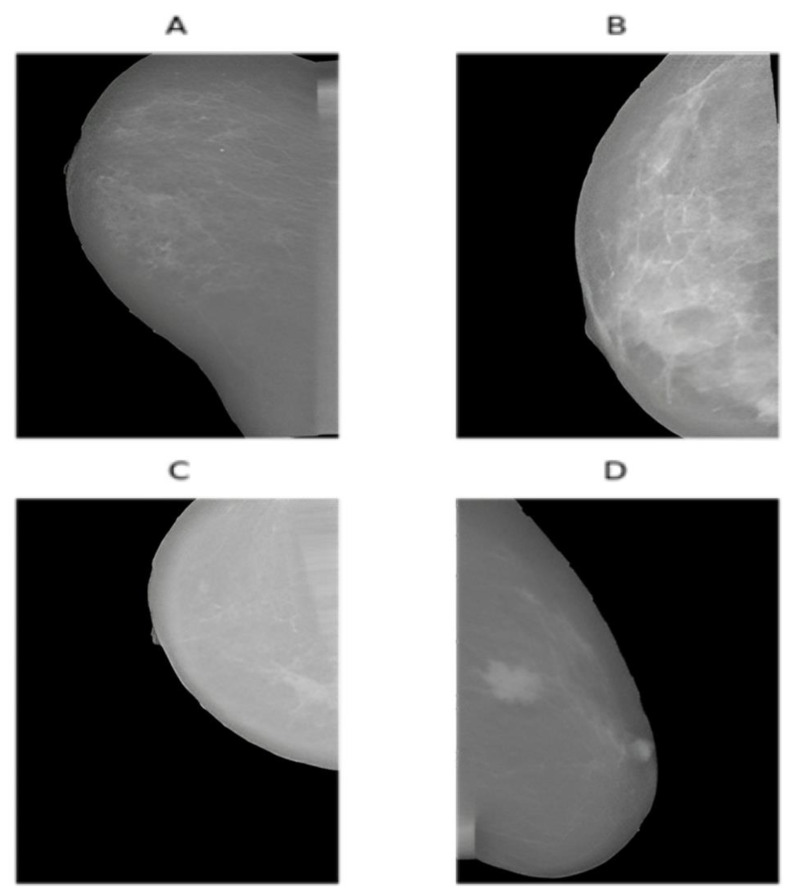
Sample augmented images from the INbreast dataset: (**A**) displays a random horizontal shift and a random vertical flip. (**B**) shows a random rotation, while (**C**) shows both a random rotation and a random vertical shift. Lastly, (**D**) displays the effect of shearing.

The INbreast dataset [53] contains 410 images, with 100 labeled as malignant and the remainder identified as benign. Following the resizing of all images to 400 × 250 pixels, we designated 20% of the dataset as test data, with the remaining 80% used for training. We then augmented both training and test data using a range of affine transformation techniques, such as rescaling, width shift, height shift, shearing, random zoom, horizontal flip, vertical flip, and random rotation. This augmentation increased our training dataset to 4000 images (inclusive of the original images), with an equal number of positive and negative cases. The same process was applied to the test data, resulting in 1000 augmented images that were equally divided between malignant and benign. Figure 3 presents the sample augmented images from the INbreast dataset.

For this study, we trained our models using the Adam [55] optimizer and binary cross-entropy loss function with a batch size of 32. We chose the Adam optimizer due to its adaptive learning rate capabilities and efficient handling of sparse gradients, making it particularly suitable for optimizing the deep neural networks employed in this study. The initial learning rate for the networks was set at 0.001. The models were trained via cross-validation with four folds.

In addition to the aforementioned strategies, we incorporated the transfer-learning technique into our model development process. Training a DL network typically requires a substantial, labeled dataset, a requirement that presents a significant challenge in medical imaging due to limited data availability. Additionally, the small size of available labeled datasets often leads to overfitting, a common issue in machine learning models. Transfer learning offers a solution to these challenges by leveraging the knowledge gained from one problem and applying it to a different but related problem. In this study, we employed transfer learning using weights derived from networks previously trained on the ImageNet dataset [56]. This approach allowed us to make effective use of our limited labeled datasets without compromising model performance due to overfitting. As for the technical aspect, all training processes were executed on an Nvidia (Nvidia Corporation, Santa Clara, CA, USA) GeForce GTX 1070 8 GB GPU. All codes were conducted using Python version 3.10 (Python Software Company, Fredericksburg, VA, USA).

## 3. Results

The performance comparison of the proposed CAW V2 method against five prominent DL networks, the majority voting decision fusion method, and the CAW V1 method are summarized in Table 1.

**Figure 4:**
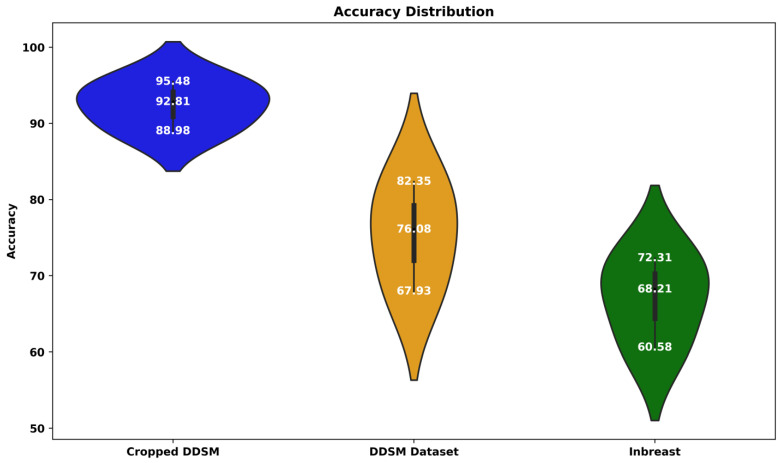
Performance comparison of various DL models on breast cancer classification: evaluating F2 scores (%).

Table 1 underscores that our proposed CAW V2 method outperforms all other models across all datasets regarding the F2 score. Specifically, our method achieved F2 scores of 95.48%, 82.35%, and 72.31% for the cropped DDSM, DDSM, and INbreast datasets, respectively. This represents an improvement of 1.59%, 1.32%, and 1.11% over the best-performing individual networks. Additionally, as the CAW V2 method is an extension of the CAW V1, it naturally exhibits superior performance. Figure 4 displays a comprehensive comparison of the individual performance of different models, along with the proposed ensemble method. The mean F2 scores for cropped DDSM, DDSM, and INbreast are determined to be 92.81, 76.08, and 68.21, respectively.

Notably, the proposed ensemble method outperforms all other methods in terms of F2 accuracy across all evaluated methodologies. These findings reinforce the superiority of the ensemble approach in achieving enhanced performance and validate its effectiveness in improving the accuracy of the aforementioned methods.

In Table 2, the calculated ‘C’ parameters of the proposed method are presented.

A cropped DDSM dataset is not a complex dataset for DL networks because it includes images that are small and easy-to-locate lesions. In contrast, the DDSM and INbreast datasets are difficult to interpret. Since images in these datasets contain the whole mammogram, finding lesions within such large images would be challenging for DL networks. According to Table 2, the more complex and difficult the dataset is, the higher the ‘C’ parameter will be. The results of our study suggest that the discrepancy between the networks’ performances in datasets can explain this phenomenon (Table 3).

Table 3 elucidates the performance variance among various DL networks across multiple datasets. Specifically, the disparity between the most efficient (EfficientNet with an F2 score of 93.89%) and the least efficient (ResNet50 with an F2 score of 88.98%) networks on the cropped DDSM dataset is notable. The ‘C’ parameter, utilized to optimize the F2 score, shows a direct correlation with these performance gaps. In this case, the performance difference of 4.9% corresponds to a ‘C’ parameter value of 2.6. However, in datasets with larger performance disparities, such as DDSM and INbreast, where the gap exceeds 10%, ‘C’ parameters greater than 3 are required.

Table 4 offers a comparison between the weights and performance improvements of our proposed CAW V1 and CAW V2.

In Table 4, we can see how the ‘C’ parameter in the CAW V2 formula affects the models’ weights. For example, in the cropped DDSM dataset, EfficientNetB3’s performance is superior to others (Table 1), so its weight increased from 0.205 to 0.213. In contrast, ResNet50 has been given a weight reduction from 0.194 to 0.185 due to its poor performance in the cropped DDSM dataset. As a result of the use of CAW V2 weights, the F2 score was improved by 0.93% in cropped DDSM, by 0.69% in DDSM, and by 0.33% in INbreast data. The open-source code is available at https://github.com/dehghanr/BreastCancer_AdaptiveWeighting (accessed on 23 October 2023).

## 4. Discussion

The primary aim of our study was to enhance early breast cancer detection by introducing a CAD ensemble system utilizing multiple advanced deep learning networks. The integrated consensus-adaptive weighting (CAW) method led to significant improvements, with the cropped DDSM dataset showcasing an accuracy boost of 95.48%. Notably, our approach also innovatively addressed pixel-level data annotation challenges in faster R-CNNs, emphasizing its potential to elevate mammogram interpretation and patient care. This research represents a significant stride toward improving breast cancer detection accuracy, potentially reducing unnecessary biopsies, and ultimately improving patient care.

In Ribli et al.’s work [33], the primary challenge with using faster R-CNN for breast cancer detection was the need for pixel-level annotations in mammogram images. This requirement is both time-consuming and costly, especially in the medical realm, where acquiring such detailed annotations demands specialized expertise and considerable resources. Chen et al. [57] further underscored this challenge by highlighting that medical image datasets often comprise fewer images compared to typical computer vision datasets, and only a fraction benefit from expert annotations. Xu et al. emphasized a similar sentiment, noting the inherent difficulty and ambiguity associated with obtaining detailed annotations for medical images [58]. They advocated for the use of models less dependent on annotated datasets, particularly spotlighting the potential of unsupervised feature learning, which can extract meaningful features without requiring labeled data. Given the inherent complexity of mammogram images, where minuscule yet crucial regions of interest are embedded in high-resolution data, the task is further magnified. Our research provides a solution to this. By introducing an ensemble system, we effectively eliminate the need for these intricate pixel-level annotations. The CAW method, which combines five distinct deep learning approaches, promises to be a more cost-effective and efficient alternative to traditional region-based networks.

Two recent studies [33,59] utilized deep learning models for breast cancer classification using FFDM images. Both studies utilized ROI annotation for breast cancer classification. In contrast, our method does not use ROI annotation. Annotating medical datasets, including mammograms [32], is particularly challenging. Compared to the existing methodologies, our results showcased consistent superiority in performance, achieving F2 scores of 95.48%, 82.35%, and 72.31% across cropped DDSM, DDSM, and INbreast datasets, respectively. This improvement, especially when ROI annotation is sidestepped, establishes our method as a significant leap forward in breast cancer classification using FFDM images. Aggregating the predictive outcomes from multiple classifiers is a prevalent strategy in machine learning [60,61]. The primary benefit of this method is that it safeguards against a solitary model being adversely influenced by outliers, noise, or complex scenarios. While individual networks such as MobileNet and Xception have been integrated for mammography [62,63], our work is pioneering in leveraging their combined strengths with an advanced weighting strategy. Distinctively, our proposed consensus-adaptive weighting (CAW) system not only fuses the decisions but also adaptively weighs them using the F2 score, emphasizing recall—a critical factor in medical diagnostics.

In our study, the nature and size of the datasets presented distinct challenges for training our deep learning model. With the cropped DDSM and DDSM datasets, their larger size offered the flexibility of random selection to mitigate potential biases. This randomness in the selection, especially given the vast number of images in the cropped DDSM dataset, ensured a balanced representation for training.

On the other hand, the INbreast dataset, with its modest count of 410 images, posed a unique challenge. Given its smaller size, the necessity for augmentation became evident to ensure adequate data for training. This augmentation, achieved through various affine transformation techniques, expanded our dataset substantially. However, it is worth noting the inherent challenges that arise with such augmented data. While these transformations increase the dataset’s diversity, they introduce images that are not “natural” in their origin. Such alterations might differ from authentic mammograms in subtle ways, potentially impacting the model’s learning dynamics. The challenge then lies in ensuring the model remains robust and generalizable despite training on these transformed images.

Our study echoes the importance of mammograms in breast lesion classification and the integral role CAD systems play in assisting medical professionals in the interpretation of such complex images. We believe that our method could be successfully extended to a larger number of DL models, opening new avenues for future research and offering significant potential for improving the early detection of breast cancer and, by extension, patient outcomes.

Despite the advancements our study offers in the realm of breast cancer detection using deep learning, there are certain limitations to acknowledge. We evaluated our model on three datasets; however, real-world applications might present unforeseen challenges, such as variations in image quality, data acquisition techniques, and differences in populations, that our controlled study environments did not account for. Moreover, while our ensemble model incorporates five different DL networks, there are numerous other potential architectures that might further improve performance. Moving forward, addressing these limitations can pave the way for an even more robust and universally applicable CAD system for breast cancer detection.

This research opens up several avenues for future exploration. One particular area of interest lies in the exploration of different mammographic datasets. The DDSM, for instance, is comprised of scanned analog film mammograms, while the INbreast database consists of FFDMs that are captured directly in a digital format. The distinction in image quality between these two types of datasets can potentially affect the performance of DL models. Researchers are encouraged to consider data augmentation techniques or amalgamating these datasets to create larger, more balanced datasets, ultimately enhancing model performance and robustness. Also, due to the flexibility and adaptability of our proposed ensemble model, CAW holds significant potential for extension beyond the realm of mammogram image analysis and DL models. Its application need not be confined to the clinical sphere. In fact, the ensemble model is equally applicable to diverse types of machine learning models, extending from decision trees and support vector machines to boosting and bagging techniques. This makes it a versatile solution for numerous predictive tasks in a myriad of research areas. Furthermore, it is important to note that while our current application is focused on image data, the model is not limited to these data types. The methodology can be readily adapted for datasets in other forms, such as numerical, categorical, or textual data. This presents an exciting opportunity for researchers to explore the adaptability and performance of our ensemble model across a spectrum of machine learning models and data types, promising significant contributions to fields beyond medical imaging.

Additionally, refining hyperparameter optimization stands out as an essential frontier in advancing the performance of our model. To achieve this, we advocate for an extensive hyperparameter optimization tailored for each deep learning model. By systematically traversing the hyperparameter space, one can pinpoint optimal configurations, thereby enhancing each model’s performance. Using cutting-edge techniques, such as Bayesian optimization or genetic algorithms, could significantly streamline this search process. Not only do these techniques locate the best configurations, but they also conserve computational resources. Moreover, it is imperative to delve into the ramifications of various hyperparameters on the ensemble’s performance. By examining elements like learning rate, batch size, and weight initialization, researchers can gain insights into how different hyperparameters impact the ensemble, offering valuable cues for subsequent model implementations. It is worth noting that our research has undertaken a comprehensive exploration of the ‘C’ hyperparameter. The possibility of utilizing a machine learning model for training this specific parameter could be explored to elevate efficiency in future endeavors. Pursuing these avenues of research promises to usher in notable progress in breast cancer detection, facilitate comprehensive performance assessments, facilitate rigorous comparative analyses, and reveal potent optimization techniques, all in service of bolstering the efficacy of our proposed CAD system.

## 5. Conclusions

Our research contributes a novel CAD system to the field of breast cancer diagnostics. This system, a collective of multiple advanced DL networks, utilizes our novel CAW method. As a result, we have observed improved breast cancer detection rates, most notably in the cropped DDSM dataset, where our system achieved an accuracy of 95.48% and increased detection rates by approximately 1.59%. We have addressed the challenge of pixel-level data annotation in faster R-CNNs and demonstrated the benefits of efficiently integrating multiple DL networks.

Our findings underscore the crucial role of mammography in breast lesion classification and illustrate the growing necessity for CAD systems in medical image interpretation. This study lays the foundation for future research in this area, indicating the potential for employing even more diverse DL models in such an ensemble-based system. The essence of our study is the delivery of an ensemble-based CAD system that significantly improves the early detection of breast cancer. By integrating various DL networks and implementing innovative adaptive weighting methods, we anticipate a progression toward more precise, swift, and patient-friendly breast cancer diagnostics.

## Figures and Tables

**Table 1:** Results of the networks and proposed weighting method for ensemble classifier.

Model	F2-Score (%)		
	Cropped DDSM	DDSM	INbreast
EfficientNet	93.89 ± 0.09	81.03 ± 0.13	63.76 ± 0.11
Xception	92.03 ± 0.05	68.06 ± 0.12	66.75 ± 0.12
MobileNetV2	92.43 ± 0.16	69.56 ± 0.28	60.58 ± 0.29
InceptionV3	91.01 ± 0.17	76.74 ± 0.35	71.20 ± 0.15
ResNet50	88.98 ± 0.16	67.93 ± 0.24	67.42 ± 0.38
Majority Vote	94.12 ± 0.12	81.32 ± 0.17	71.68 ± 0.16
Initial proposed method: CAW V1	94.55 ± 0.10	81.66 ± 0.18	71.98 ± 0.15
Final proposed method: CAW V2	95.48 ± 0.08	82.35 ± 0.17	72.31 ± 0.16

Mean ± std. CAW: consensus-adaptive weighting method. DDSM: Digital Database for Screening Mammography dataset. V1: Version 1. V2: Version 2.

**Table 2:** Final ‘C’ values of the proposed CAW V2 method according to the type of dataset.

C (CAW V2 Formula)		
Cropped DDSM	DDSM	Inbreast
2.6	3.4	3.1

CAW: Consensus-adaptive weighting method. DDSM: Digital Database for Screening Mammography dataset. V2: Version 2.

**Table 3:** Differences between the best and the worst DL networks’ performances in different datasets.

	Dataset		
	Cropped DDSM	DDSM	INbreast
Best and worst performance differences (%)	4.906	13.105	10.62

DDSM: Digital Database for Screening Mammography dataset.

**Table 4:** Comparison of CAW V1 and V2.

Model	Comparison of CAW V1 and V2					
	Cropped DDSM		DDSM		INbreast	
	V1 Weights	V2 Weights	V1 Weights	V2 Weights	V1 Weights	V2 Weights
EfficientNet B3	0.205	0.213	0.223	0.283	0.193	0.178
Xception	0.201	0.202	0.187	0.156	0.202	0.191
MobileNetV2	0.202	0.204	0.191	0.148	0.184	0.135
InceptionV3	0.199	0.196	0.211	0.186	0.216	0.202
ResNet50	0.194	0.185	0.187	0.151	0.204	0.214
CAW model (F2 score % Improvement)	0.93 ± 0.18		0.69 ± 0.35		0.33 ± 0.31	

V1: Version 1, V2: Version 2, V3: Version 3, CAW: consensus-adaptive weighting method.

## Data Availability

Here are the publicly available links to the datasets we utilized: cropped DDSM dataset download link: https://www.kaggle.com/skooch/ddsm-mammography (accessed on 5 February 2021); DDSM dataset download link: https://www.kaggle.com/datasets/cheddad/miniddsm2 (accessed on 18 March 2021); INbreast dataset download link: https://www.kaggle.com/datasets/ramanathansp20/inbreast-dataset?resource=download (accessed on 3 March 2021).
